# Risk Factors and Impact on Survival of Pathological Fractures in Patients With Humeral Metastasis

**DOI:** 10.7759/cureus.73382

**Published:** 2024-11-10

**Authors:** Shiro Saito, Hiroaki Kimura, Hisaki Aiba, Yohei Kawaguchi, Hideki Murakami

**Affiliations:** 1 Department of Orthopaedic Surgery, Nagoya City University Graduate School of Medical Sciences, Nagoya, JPN

**Keywords:** bone modifying agents, chemotherapy, humeral metastasis, overall survival, pathological fracture

## Abstract

Background

Bone metastases often cause pathological fractures and impair patients’ quality of life and survival. Although several studies have been conducted on pathological fractures in the femur and spine, limited research has been done on the upper limbs. This study aimed to reveal the risk factors and determine how pathological fractures impact survival in patients with humeral metastasis.

Methods

This retrospective study was based on patients with humerus metastasis treated in the Nagoya-City University Hospital from 2010 to 2020. Patient characteristics, including sex, age at diagnosis of humeral metastasis, primary cancer, prior treatment, anatomical location, and metastatic lesion size, were retrieved from medical records. The patients were divided into pathological fracture and non-fracture groups, and their backgrounds and survival rates were compared.

Results

Of the 31 patients with 32 metastatic lesions included in this study, 19 had pathological fractures (one patient had bilateral fractures) and 12 had no fractures. Our analysis revealed that the risk factors for pathological fracture were treatment without bone-modifying agents, treatment without radiotherapy, and larger circumferential cortical involvement. The median overall survival was 21 months; 1-year survival was 56% in the non-fracture group and 59% in the fracture group. There was no significant difference in survival rates between the two groups and only chemotherapy correlated with longer survival in multivariate analysis.

Conclusions

Bone-modifying agents have the benefit of preventing pathological fractures due to humeral metastases. The humeral pathological fracture did not affect the patient's survival, and chemotherapy was the only prognostic factor that prolonged survival.

## Introduction

Recent advances in cancer treatment have improved the survival of patients with cancer, but the frequency of bone metastases has also increased. They often cause pathological fractures and impair patients’ quality of life and survival [1]. Therefore, it is necessary to predict pathological fractures and provide interventions for patients at high risk. Mirels reported a scoring system that predicts the risk of pathological fractures based on the location of bone metastasis, degree of pain, radiographic findings, and lesion size and suggested that surgical intervention should be considered if the score is 9 or higher [2]. However, the Mirels score has low inter-observer concordances, and the lack of specificity possibly induces overtreatments [3]. Van der Linden et al. reported that axial cortical involvement >30 mm and circumferential cortical involvement >50% were predictive factors of a pathological fracture due to femoral metastasis [4]. Although there are other studies on pathological fractures in the femur and spine, currently, there is limited research on the upper limbs.

Fracture prevention includes bone-modifying agents, radiation therapy, and surgery. These are considered based on the patient's prognosis and the risk of complications. In the case of the femur, surgery before pathological fracture is superior to that after fracture in terms of post-operative function [5]. In addition, post-operative walking function is related to survival time in femoral and spinal metastases [6,7]. Maintaining walking function and performance status is thought to lead to continued chemotherapy and prolonged survival. On the other hand, pathological fractures in the upper limbs do not always affect walking function or performance status, and the impact on survival is unknown. The purpose of this study was to identify the risk factors and impact on the survival of pathological fractures in patients with humeral metastasis, the most frequently affected bone in the upper limbs.

## Materials and methods

**Figure 1:**
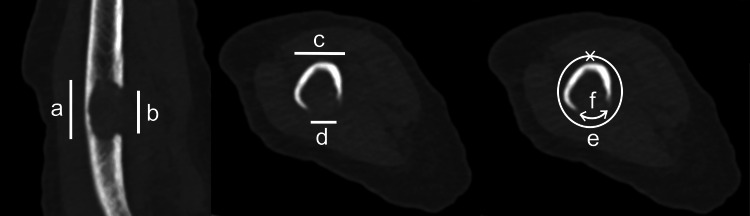
Images of measuring bone lesions a: Height (mm)　b: Axial cortical involvement (mm)　d/c: Maximum diameter (%)　f/e: circumferential cortical involvement (%) The maximum height was measured in the craniocaudal plane, and the maximum diameter was measured in the transverse plane. Axial cortical involvement is the length of cortical lysis in the craniocaudal plane. Circumferential cortical involvement was determined by calculating the ratio of circumferential cortical lysis to the bone perimeter in the most at-risk area.

We included patients who were diagnosed with humeral metastasis of solid tumors or hematological malignancies from 2010 to 2020 in Nagoya City University Hospital. Patient characteristics, including sex, age at diagnosis of humeral metastasis, primary cancer, prior treatment (chemotherapy, bone-modifying agents, or radiotherapy to the metastatic site), and the anatomical location and the size of the metastatic lesion, were retrieved from the medical records. The anatomical location was classified as proximal, diaphyseal, or distal of the humerus. Radiological evaluation of metastatic lesions was performed using computed tomography to measure maximum height, diameter, axial cortical involvement, and circumferential cortical involvement (Figure 1). 

The maximum height was measured in the craniocaudal plane, and the maximum diameter was measured in the transverse plane. Axial cortical involvement was mentioned by van der Linden et al. [8], which is the length of cortical lysis in the craniocaudal plane. Circumferential cortical involvement was determined by calculating the ratio of circumferential cortical lysis to the bone perimeter in the most at-risk area. The patients were divided into the pathological fracture and non-fracture groups, and their backgrounds and survival rates were compared. In addition, in cases of pathological fractures, the efficacy of surgery on clinical outcomes was evaluated.

The statistical analysis was conducted using the Mann-Whitney U test and Fisher's exact test for the univariate analysis. Logistic regression analysis was used for the multivariate analysis. The threshold of the lesion size for fracture was analyzed using a receiver operating characteristic curve. The overall survival curves were calculated using the Kaplan-Meier method. A Log-Rank test was used to compare the survival curves. The Cox-proportional hazard model was used for the calculation of survival over time at a 95% confidence level. The value of p was considered significant when <0.05. All statistical analyses were performed with EZR (Saitama Medical Center, Jichi Medical University, Saitama, Japan), which is a graphical user interface for R (The R Foundation for Statistical Computing, Vienna, Austria). More precisely, it is a modified version of R commander designed to add statistical functions frequently used in biostatistics [9].

## Results

In total, 31 patients were included in this study (Table 1).

**Table 1:** Patient characteristics and comparison of the fracture and non-fracture group The statistical analysis was conducted using the Mann–Whitney U test or Fisher's exact test for the univariate analysis. Logistic regression analysis was used for the multivariate analysis. NS: not significant.

	Fracture	Non-fracture	Univariate Analysis	Multivariate Analysis
Number of lesions (n, %)	19 (61.3%)	12 (38.7%)		
Follow-up after diagnosis (median and range in months)	10 (0.5-91)	12 (3-92)	NS (p=0.49)	NS (p=0.899)
Age (median and range in years)	67 (51-85)	65.5 (44-81)	NS (p=0.416)	NS (p=0.208)
Sex				
Male (n, %)	10 (32.2%)	6 (19.4%)	NS (p=1)	NS (p=0.781)
Female (n, %)	9 (29.0%)	6 (19.4%)		
Primary cancer				
Lung (n, %)	5 (16.1%)	6 (19.4%)	NS (p=0.255)	NS (p=0.988)
Hematologic malignancies (n, %)	5 (16.1%)	2 (6.5%)	NS (p=0.676)	NS (p=0.417)
Kidney (n, %)	4 (12.9%)	1 (3.2%)	NS (p=0.624)	NS (p=0.922)
Breast (n, %)	2 (6.5%)	2 (6.5%)	NS (p=0.630)	NS (p=0.920)
Others (n, %)	3 (9.7%)	1 (3.2%)	NS (p=1)	
Anatomical location				
Proximal (n, %)	4 (12.9%)	6 (19.4%)	NS (p=0.127)	NS (p=0.078)
Diaphyseal (n, %)	15 (48.4%)	6 (19.4%)		
Chemotherapy				
Yes (n, %)	12 (38.7%)	11 (35.5%)	NS (p=0.108)	NS (p=0.415)
No (n, %)	7 (22.6%)	1 (3.2%)		
Bone modifying agents				
Yes (n, %)	3 (9.7%)	8 (25.8%)	p=0.007	p=0.029
No (n, %)	16 (51.6%)	4 (12.9%)		
Radiotherapy				
Yes (n, %)	6 (19.4%)	10 (32.3%)	p=0.025	NS (p=0.143)
No (n, %)	13 (41.9%)	2 (6.5%)		
Dimensions (median and range)				
Maximum height (mm)	50 (24-109)	39 (12-138)	NS (p=0.123)	NS (p=0.728)
Axial cortical involvement (mm)	29 (6-91)	24 (6-37)	NS (p=0.084)	NS (p=0.604)
Maximum diameter (%)	89 (65-100)	74 (43-89)	NS (p=0.073)	NS (p=0.221)
Circumferential cortical involvement (%)	63 (16-84)	24 (7-72)	p=0.004	p=0.046

**Figure 2:**
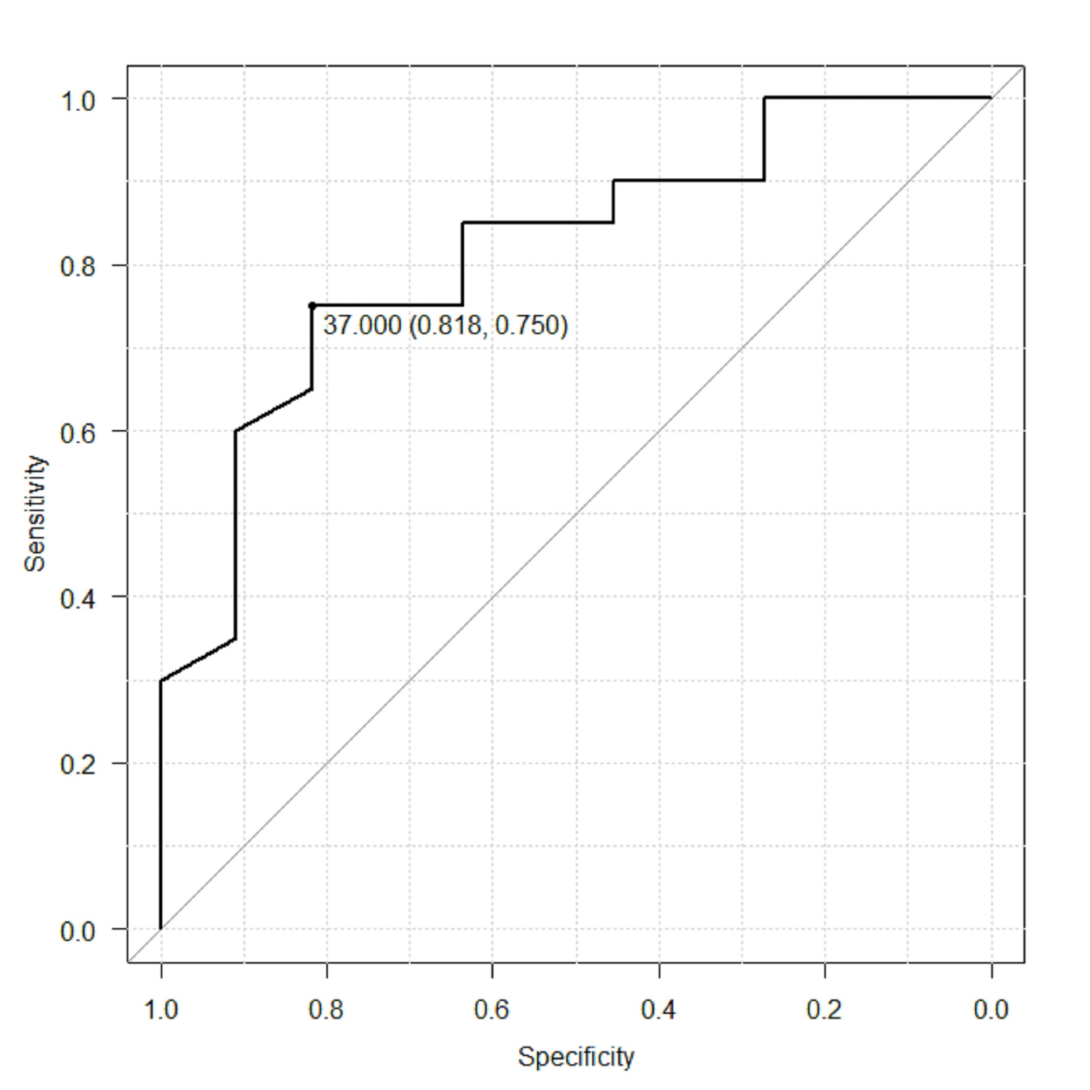
Receiver operating characteristic curve of the circumferential cortical involvement threshold 37% threshold: sensitivity = 75%, specificity = 81.8%

The median follow-up was 11 months after the diagnosis of humeral metastases. Nineteen patients had pathological fractures, and 12 patients had no fractures. In the fracture group, 10 patients (53%) had a fracture at the time of diagnosis, and 9 patients (47%) presented at a median time of 1 month (1 week to 3 months) after diagnosis of bone metastasis. The most common primary tumor was that of the lungs (35%) followed by hematologic malignancies including multiple myeloma and lymphoma (23%), kidney (16%), breast (13%), and others (stomach, esophagus, liver, and cervix). The anatomical locations of bone metastases were proximal in 32% of the cases and diaphyseal in 68% of the cases. None of the patients had metastases in the distal humerus. There was no statistically significant difference in sex, age, primary cancers, and anatomical locations between the fracture group and non-fracture group. The risk factors of pathological fracture were treatment without bone modifying agents (p=0.007), without radiotherapy (p=0.025), and larger circumferential cortical involvement (p=0.004) in univariate analysis. In multivariate analysis, using bone modifying agents reduced the risk of fracture (odds ratio=0.0191, 95% confidence interval 0.000556-0.66, p=0.029) and larger circumferential cortical involvement was the predictive factor for fracture (odds ratio=1.07, 95% confidence interval 1.00-1.14, p=0.046). The threshold of circumferential cortical involvement that indicates the maximum sensitivity and specificity for pathological fracture was 37% (Figure 2).

**Figure 3:**
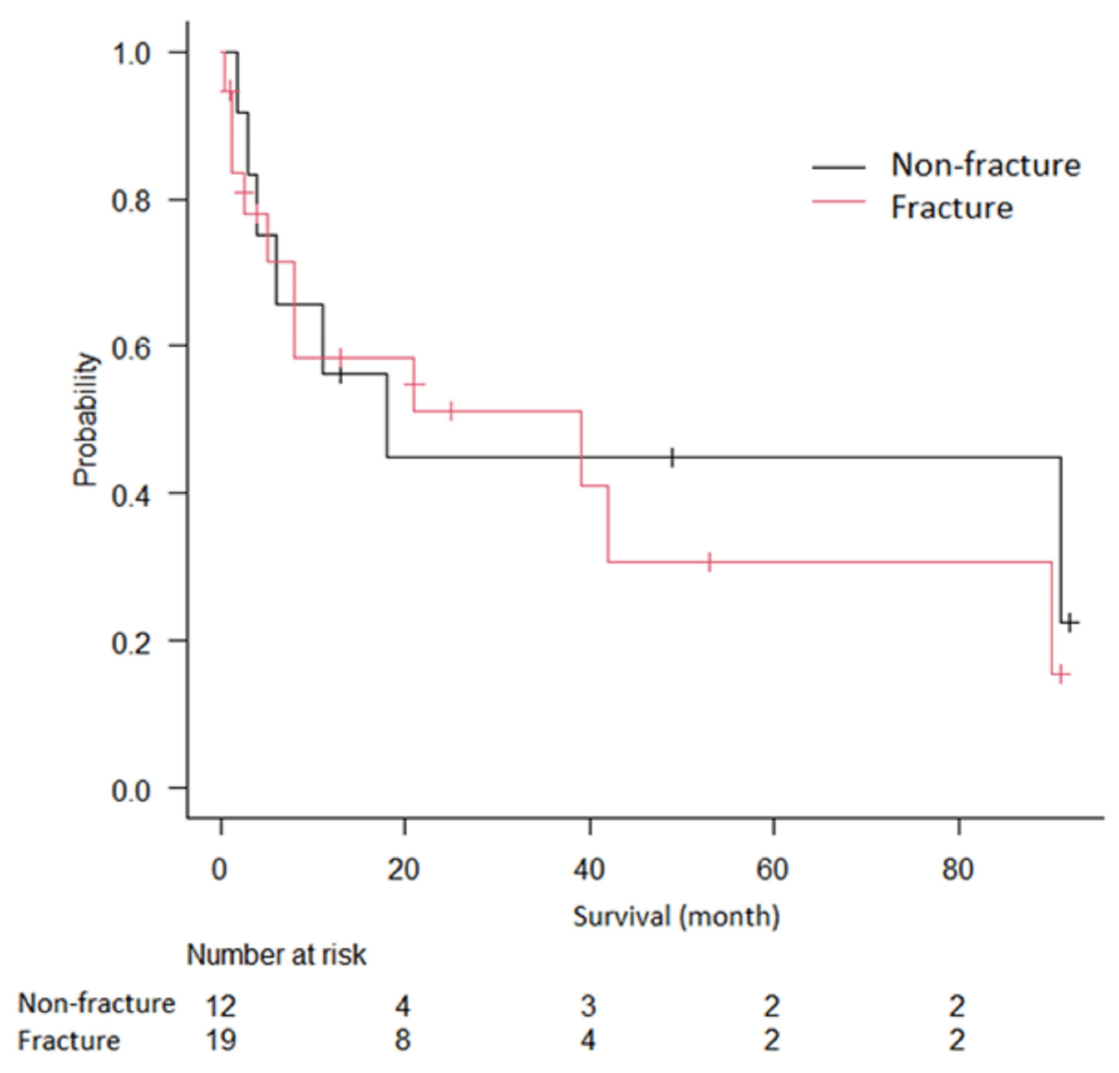
Kaplan–Meier curve of overall survival in the fracture group and non-fracture group. There was no significant difference in survival rate between the two groups.

The median overall survival was 21 months; 1-year survival was 56% in the non-fracture group and 59% in the fracture group. There was no significant difference in survival rate between the two groups (Figure 3), and chemotherapy had a correlation with longer survival in multivariate analysis (hazard ratio=0.03, 95% confidence interval 0.001-0.945, p=0.046).

In 19 patients with pathological fractures, four patients underwent surgery, and 15 underwent conservative treatment. The median overall survival was 21.5 months (1-90) in the surgical group, and 10 months (0.5-91) in the conservative group. In 10 patients with a follow-up of 6 months or more, the surgical group tended to have a better range of motion and required fewer analgesics than the conservative group (Table 2).

**Table 2:** The characteristics of patients with pathological fracture followed up for over 6 months B: Bone Modifying Agents　C: Chemotherapy　R: Radiotherapy

Age (y)	Sex	Primary cancer	Surgery for fracture	Other treatment	Follow up (month)	Range of motion (best)	Analgesics use
73	Female	Lung	No	R	20	No rehabilitation	Celecoxib 100 mg×2
51	Female	Breast	No	C+R	8	No rehabilitation	Loxoprofen 60 mg×3
79	Male	Kidney	No	C+R	16	Abduction 90	Loxoprofen 60 mg×3
71	Male	Lung	No	B+C	12	No rehabilitation	Acetoaminophen 400 mg×3
80	Female	Myeloma	No	B+C	25	Anterior elevation 90	Oxycodone 5 mg
60	Male	Kidney	No	B+C	49	No rehabilitation	None
82	Female	Myeloma	No	B+C	24	Anterior elevation 60	Tramadol 25 mg×5
58	Male	Lung	No	B+C+R	13	Abduction 90	Oxycodone 20 mg
76	Female	Breast	Yes	B+C	39	Anterior elevation 150	None
61	Female	Kidney	Yes	B+C	90	Anterior elevation 170	Acetoaminophen 200 mg×3

## Discussion

The humerus is the second most frequently involved long bone of metastasis, following the femur [10]. Although there are numerous studies on pathological fractures in the femur, there is still limited research on pathological fractures in the humerus. In this study, we focused on humeral metastasis and analyzed fracture risk and overall survival. Although the fracture rate of long bones due to metastasis was reported to be 10-29% [11], our study reported a 61% fracture rate in humeral metastasis. Hoban et al., also reported that the overall fracture rate was 76% in 45 patients with upper limb bone metastases [12]. The high fracture rate is thought to be due to the fact that bone metastases in the upper limbs are not indicated for prophylactic fixation, unlike those in the lower extremities, which directly impair the patient's walking function and performance status. Moreover, upper limb metastases are difficult to detect early because they cause less pain than lower limbs. Approximately 53% of patients in this study had no detected humeral metastases until the fracture occurred.

The risk factor of humeral pathological fracture was treatment without bone-modifying agents in multivariate analysis. Eleven patients were treated with bone-modifying agents (four zoledronic acid and seven denosumab) in this study, and three patients had pathological fractures. While the efficacy of zoledronic acid and denosumab for bone metastasis from solid tumors or multiple myeloma has been demonstrated in many studies to date [13-16], we showed that they also reduce pathological fracture risk in humeral metastasis.

Radiotherapy is known to relieve pain caused by bone metastases, especially in radiosensitive tumors (e.g. hematologic malignancies, breast and prostate cancers), and should be suggested to patients [17]. However, the efficacy of radiotherapy for preventing pathological fractures is still unclear, and Tatar et al. reported that a circumferential cortical involvement of >30% induced pathological fractures at a high rate after radiotherapy [18]. Therefore, for patients with a high risk of fracture, it is preferable to use radiotherapy in combination with other treatments (surgery or bone-modifying agents).

Regarding the size of metastatic lesions, our study suggested that a circumferential cortical involvement >37% (sensitivity and specificity were 75% and 81.8%, respectively) was a predictive factor for humeral pathological fractures. On the other hand, there was no significant difference in axial cortical involvement. Van der Linden et al. reported that an axial cortical involvement of >30 mm and a circumferential cortical involvement of >50% are predictive factors for a pathological fracture in the femur [4]. Although the humerus has a thinner cortical thickness than the femur, it receives rotational forces more frequently than axial pressures, potentially supporting this result. Still, our study had a small sample size, and further research is required for an accurate cut-off value.

In addition, our study showed that chemotherapy was the only prognostic factor, and humeral pathological fracture did not affect patient survival. In a retrospective analysis of 102 patients with upper limb bone metastases, Wisanuyotin et al. found that the significant risk factors for survival were the type of primary tumor and ECOG performance status, and did not include pathological fractures [19]. Pathological fractures in the femur and spine have been reported to shorten patient survival as it becomes difficult to continue chemotherapy due to worse walking function and performance status. However, pathological fractures in the upper extremities do not necessarily impair performance status, and chemotherapy can be continued after the fracture. In this study, 84% of patients underwent chemotherapy after fracture, and the overall 1-year survival was 58%. Wedin et al. reported that the 1-year survival of 208 patients after surgery for humeral metastases was 40% [20]. Our results are superior to those from previous studies, and survival predictions should be updated as treatments advance.

Surgical treatment for pathological fractures is generally determined by the prognosis of the cancer patient. If the patient's prognosis for survival is expected to be more than 3 months and their general condition is stable, surgery may be considered. Although our study had a small number of patients who underwent surgery, they tended to have a better range of motion and less pain than patients who underwent conservative treatment. Previous reports have also shown that surgery for pathological humeral fractures is effective in terms of pain relief and functional recovery [21,22], which is consistent with our results. However, as it often requires interruption of chemotherapy, the treatment for humeral metastasis should be considered on a case-by-case basis.

Our study had several limitations. This was a retrospective study conducted at a single institution with a small sample size. In addition, as the responses to chemotherapy can vary considerably among patients because of differences in the primary tumor, larger studies need to be conducted to validate our findings.

## Conclusions

Our study suggested that bone-modifying agents have the benefit of preventing pathological fractures due to humeral metastases, and larger circumferential cortical involvement was a predictive factor for pathological humeral fractures. However, the humeral pathological fracture did not affect patient survival, and chemotherapy was the only prognostic factor that prolonged survival. 
